# Routine whole body CT of high energy trauma patients leads to excessive radiation exposure

**DOI:** 10.1186/s13049-016-0199-2

**Published:** 2016-01-27

**Authors:** Fredrik Linder, Kevin Mani, Claes Juhlin, Hampus Eklöf

**Affiliations:** Department of Surgical Sciences, Section of Vascular Surgery, Uppsala University, Uppsala, Sweden; Department of Surgical Sciences, Section of Radiology, Uppsala University, Uppsala, Sweden

## Abstract

**Background:**

Whole body computed tomography (WBCT) is an important adjunct in trauma care, which is often part of standard protocol in initial management of trauma patients. However, WBCT exposes patients to a significant dose of radiation. The use of WBCT was assessed in a modern trauma cohort in Sweden.

**Methods:**

A two-center retrospective cohort study was performed. All consecutive trauma alert patients at a university hospital (July-December 2008), and a rural county hospital (January 2009- December 2010) were included. Patients were stratified into three groups (high, intermediate and low risk) based on documented suspected injuries at primary survey at the site of accident or at the emergency department. Injury severity score (ISS) was calculated. Case records were reviewed for clinical and radiological findings at the time of trauma, and during a ≥36 months of follow-up period to identify possible missed injuries.

**Results:**

A total of 523 patients were included in the study (university hospital *n* = 273; rural county hospital *n* = 250), out of which 475 patients (91.0 %) underwent radiological examinations, 290 patients (55.4 %) underwent WBCT, which identified trauma related findings in 125 patients (43.1 % of those examined). The high-risk group (*n* = 62) had a mean age of 38.5 years (21.1 SD). Mean ISS was 16.48 (18.14 SD). In this group, WBCT resulted in a positive finding in 38 (74.5 %) patients. In the intermediate-risk group (*n* = 322; mean age 37.66, 20.24 SD) ISS was 4.42 (6.30 SD). A positive finding on WBCT was found in 87 of the intermediate group patients (44.8 %). The low-risk group (*n* = 139; mean age 32.5 years; 21.4 SD) had a mean ISS of 0.84 (1.57 SD) with no positive findings on WBCT and no missed injuries in medical records at ≥36 months.

**Discussion:**

The risk of developing radiation induced cancer is significant for young people if exposed to relatively high dose radiation as is the case in WBCT. WBCT in high-energy trauma is important for planning of treatment in severely injured patients while it can be questioned in the seemingly not injured where it is used mainly to permit early discharge from the ED.

**Conclusions:**

Risk stratification criteria could in this retrospective study identify high energy trauma patients not in need of radiological imaging. WBCT in high-energy trauma does not affect patient care if the patient is mentally alert, not intoxicated nor shows signs of other than minor injuries when evaluated by a trauma-team. The risk of missing important traumatic findings in these patients is very low. Observation of the patient with reexamination instead of imaging may be considered in this group of often young patients where radiation dose is an issue.

## Background

The 2014 report from the European Injury Data Base shows accidents and violence to result in an annual 40 million people being treated in hospitals and 233,000 people die as a consequence [1]. Over the last decades trauma care has improved with implementation of multidisciplinary trauma teams, standardized care including implementation of advanced trauma life support (ATLS®) and the technical developments in imaging [2]. Triage and triage criteria are debated with the aim to identify those patients who benefit from activation of a trauma team and consequent rapid expedition in the Emergency department (ED). Clinical examination is often complemented with radiological imaging in the trauma room of chest and pelvis, followed by a whole body computed tomography (WBCT) for trauma. The goal is to discover serious injuries and their extent, as soon as possible, to decide on the need for life saving procedures.

Triage based trauma-alert protocols facilitate early discovery of patients who are seriously injured and consequently benefit the most from rapid care by the trauma team. The definition of serious injury is Injury Severity Score (ISS) > 15 [3], but for obvious reasons, this scoring is only available when the full extent of injuries is known. Still, triage based trauma-alert protocols aim to balance the disadvantage of overtriage leading to overuse of resources and undertriage leading to failure in recognizing patients in need of the trauma team. Standard protocol of care for trauma patients often include CT imaging. WBCT provides detailed information on known and occult injuries [4] and was implemented in the mid 90′s as a routine procedure in the Swedish health care system [5]. Sweden was an early-adopter of WBCT in trauma care, and the use of WBCT as an adjunct has been well established for 20 years. In severely injured patients WBCT will with high accuracy describe the extent of injuries. WBCT is also used to exclude occult injuries in the seemingly not injured high energy trauma patients [6]. With present routines, most trauma patients exposed to high energy trauma undergo WBCT. The rationale for WBCT is based on studies indicating high risk of clinically significant missed injuries in trauma patients [7, 8]. Imaging comes with a risk as WBCT exposes the patient to a dose of ionizing radiation >20 mSv. Exposition to ionizing radiation of this magnitude when 40 years or younger may induce cancer in 1/1000 patients [7].

In recent years there has been a shift from indiscriminate referral to WBCT of trauma patients exposed to high energy trauma, to a more risk/benefit-oriented approach suggesting clinical prediction rules to safely omit WBCT [8]. Some studies point out that WBCT can safely be omitted if certain criteria are fulfilled [9]. Evidence based guidelines may limit radiation exposure and reduce cost in a safe way [10].

The aim of this study was to evaluate if the indication for WBCT can be restricted in subgroups of trauma patients without risking clinical safety.

## Methods

A two-center retrospective cohort study was performed. All consecutive trauma alert patients at a university hospital (July-December 2008), and a rural county hospital (January 2009- December 2010) were included. Patients without a correct personal identification number were excluded due to lack of follow-up data. The patients were identified from the manual trauma register kept in the ED of the two hospitals during this time-period.

The university hospital is an urban trauma center for 360,000 inhabitants, a regional trauma referral center and a national referral center for burns and maxillofacial trauma. Its annual ED census is approximately 60,000 adult patients and major trauma census is approximately 700 patients, all referrals excluded. The county hospital is situated on an island with 57,000 inhabitants. The population of the island increases to approximately 200,000 during the summer months.

All patients were treated according to the Advanced Trauma Life Support guidelines (ATLS®). The university hospital uses a two-tiered trauma alarm protocol.

A full trauma team is called for patients with compromised physiology or type specific injuries according to Table 1. A limited trauma team is called for patients subject to high-energy trauma without compromised physiology or type specific injuries according to Table 1. The county hospital has a common trauma team for all trauma alarm based on physiology, type specific injuries and high-energy trauma according to Table 1.

**Table 1:**
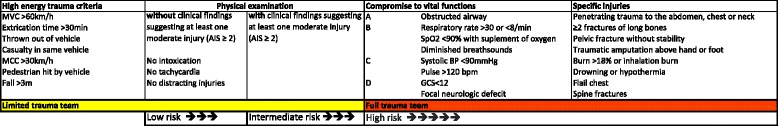
Trauma alert guidelines and risk group assessment. Regionally modified criteria for trauma alert calls, and risk group assessment

The full trauma team at the university hospital consists of the trauma leader (TL), an experienced general surgeon, a junior general surgeon, anesthesiologist, anesthesiology nurse, neurosurgeon, thoracic surgeon, orthopedic surgeon, radiologist and a radiographer, 2 ED nurses and a kin supporter. A pediatric surgeon is included when applicable. The limited trauma team at the university hospital consists of a general surgeon and 2 ED nurses. An experienced general surgeon (TL), radiologist and a radiographer are also notified, but do not attend unless warranted. The trauma team at the county hospital is coherent with the limited trauma team at the university hospital with addition of radiologist, radiology nurse, an anesthesiologist and an anesthesiology nurse.

On admission to E.D, all trauma patients receive an evaluation of the trauma team, including laboratory tests (hematocrit, blood alcohol level, urine analysis and pregnancy test when applicable), ECG and monitoring of vital parameters. Plain radiographs of chest and pelvis are taken when deemed necessary. In this study, patients who activated a full, or limited, trauma team were retrospectively divided into 3 groups based on risk of injury on first evaluation:

1. High risk – Patients with signs of compromise to vital functions or predefined injury types, Table 1.
2. Intermediate risk – Patients without signs of compromise to vital functions or predefined injury types, but with clinical findings suggesting at least one moderate injury (AIS ≥ 2), or intoxication.
3. Low risk – Patients with clinical findings limited to minor injuries (AIS ≤ 1) and no intoxication.

The risk stratification between high and intermediate risk was based on the American College of Surgeons Committee on Trauma guidelines for full and limited trauma team alerts. These guidelines have been used for the last decade in our hospitals with some local adaption. The guidelines were essentially the same at both hospitals. The stratification between intermediate and low risk was based on the first clinical exam in the E.D.

Almost all high-risk patients undergo a WBCT unless they are transferred to the operation theatre without further delay, whereas intermediate- and low-risk patients undergo WBCT or other radiological imaging if the surgeon on call deems it of value.

The WBCT was performed according to protocol starting with AP-scout, followed by native phase head and neck CT, followed by intravenous contrast-medium injection and CT of the thorax, abdomen, pelvis and upper legs in venous phase. At the University hospital an additional topogram after 5 min a was performed to evaluate kidney function by presence of renal excretion. We did not scan the patient in arterial phase unless warranted.

Injury severity was graded using the Abbreviated Injury Severity Score (AIS). This score is based on the topography and severity of a lesion. Every anatomical lesion is described on a scale from 1–6 for severity. The scale ranges from 1 (minor) to 6 (lethal). The AIS helps calculating the Injury Severity Score (ISS) [3]. The ISS is the sum of the square of the three highest AIS within separate body areas. Any lesion with AIS of 6 will automatically lead to an ISS of 75. Patients with an ISS above 15 are considered as severely injured patients.

The included patients medical records were reviewed and information extracted regarding patient age at admission, gender, mechanism of injury, findings at clinical examination, use of radiological imaging (WBCT-T and/or others), findings at radiological imaging, changes in radiological reports (preliminary vs final report), accidental findings, blood alcohol level, operations, intensive care, admission and follow-up. We used several parallel and overlapping approaches to identify and classify the outcome variables, including retrieving and evaluating all patient records from repeat or follow-up visits from the time of initial observation through December 2013 (a minimum of 36 months follow-up period). The three groups were assessed with regards to patient age and sex, mechanism of injury, the use of WBCT, radiological findings, ISS and admission to surgical ward or intensive care unit (ICU) and mortality. Subgroup analyses were performed based on mechanism of injury, admitting hospital and on patients examined with WBCT.

The study was approved by the local ethics committee (Dnr 2014–250).

### Statistics

Data were assessed for normality with histograms. Normally distributed continuous data are reported as means with standard deviation (SD), and are compared with student’s t-test. Categorical data are reported as ratios with 95 % confidence intervals (CI), and were assessed with chi-square. Study groups were compared with confidence intervals. Statistical analyses were performed with IBM SPSS Statistics version 22 (IBM Corp, Armonk, New York, USA).

## Results

A total of 523 patients were included. Eight patients were excluded; 3 due to de-activation of trauma alert; 5 patients could not be identified retrospectively.

**Fig. 1:**
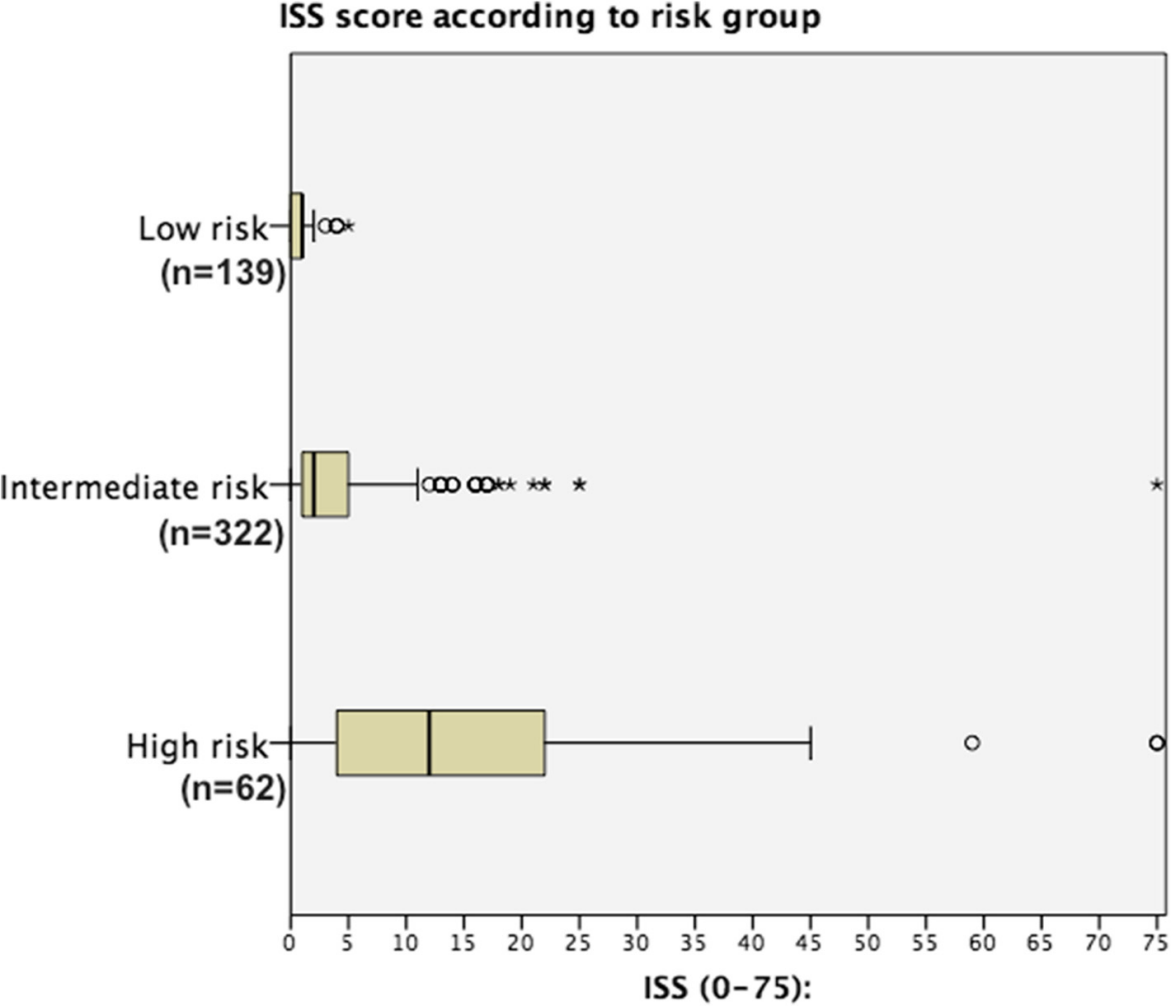
Boxplot of ISS according to risk group. After subdivision into the studied groups

The mechanism was blunt trauma in 517 patients (98.9 %). Patient characteristics are shown in Table 2 and the ISS score of the different risk groups is presented in Fig. 1. The mechanisms of injury, and their distribution in the groups are shown in Table 3. There were 229 motor vehicle collisions (MVC) with a mean ISS of 3.53 (9.62 SD) and 163 patients (71.1 %) had no significant injuries (ISS ≤ 1). Forty fall injuries from >3 m with a mean ISS of 7.95 (6.68 SD) were identified, and 9 (22.5 %) had no significant injuries (ISS ≤ 1). Patient characteristics and findings of injury were similar at the county hospital and the university hospital, Table 4. The use of imaging in trauma differed, with a higher rate of WBCT at the university hospital, counterbalanced by a higher rate of single organ radiological imaging at the county hospital. There was however no difference in rate of injury findings, Table 4. More patients were admitted for observation or treatment at the county hospital, with a large discrepancy in intensive care admissions (58 % county hospital vs 14.3 % university hospital, *p* < 0.001).

**Table 2:** Patient characteristics according to risk groups. After subdivision into the studied groups

	Low risk group	Intermediate risk group	High risk group	*P*-value
	(*n* = 139)	(*n* = 322)	(*n* = 62)	
Age - mean, years (SD)	32.52 (21.35)	37.66 (20.24)	38.49 (21.13)	0.035
Male gender % (n):	61.2 % (85)	64.3 % (207)	77.4 % (48)	0.075
Mechanism of injury % (n)				<0.001
Unprotected traffic victim (MC, bike, pedestrian)	20.9 % (29)	34.2 % (110)	9.7 % (15)	
Protected traffic victim (MVC)	62.6 % (87)	36.3 % (117)	10.9 % (25)	
Fall < 3 m	10.8 % (15)	13.4 % (43)	3.3 % (2)	
Fall > 3 m	2.9 % (4)	7.8 % (25)	27.5 % (11)	
Other (chrush, blunt, SW)	2.9 % (4)	83.9 % (27)	22.5 % (9)	
Examined with whole body computer tomography in trauma (WBCT-T) % (n)	32.4 % (45)	60.2 % (194)	82.3 % (51)	<0.001
Injury on WBCT-T % (n)	0 % (0)	44.8 % (87)	74.5 % (38)	<0.001
Specific radiological exams % (n)	46.8 % (65)	58.4 % (188)	43.5 % (27)	0.016
Injury Severity Score (ISS) - mean (SD)	0.84 (1.57)	4.42 (6.30)	16.48 (18.14)	<0.001
S-etanol in mmol/l in intoxicated patients - mean (SD)	-	43.53 (23.54)	38.99 (15.80)	0.53
Intoxication on admission % (n)	0 % (0)	7.8 % (23)	25.0 % (14)	<0.001
Admission to intensive care unit (ICU) % (n)	20.1 % (28)	33.5 % (108)	77.4 % (48)	<0.001

**Table 3:** Patient characteristics according to mechanism of injury. After subdivision into the studied groups

	Unprotected traffic victim	Protected traffic victim	Fall <3 m	Fall >3 m	Other (chrush, blunt, SW)	*P*-value
	(*n* = 154)	(*n* = 229)	(*n* = 60)	(*n* = 40)	(*n* = 40)	
Age - mean, years (SD)	33.62 (20.58)	34.98 (19.18)	44.45 (25.78)	43.26 (19.15)	36.13 (19.54)	0.002
Male gender % (*n*):	64.3 % (99)	57.5 % (132)	63.3 % (38)	95.0 % (38)	82.5 % (33)	<0.001
Examined with whole body computer tomography in trauma (WBCT-T) % (*n*)	60.4 % (93)	51.5 % (118)	50.0 % (30)	72.5 % (29)	50.0 % (20)	0.065
Injury on WBCT-T % (*n*)	47.3 % (44)	28.8 % (34)	50.0 % (15)	72.4 % (21)	60.0 % (12)	<0.001
Specific radiological exams % (*n*)	61.7 % (95)	46.3 % (106)	56.4 % (34)	62.5 % (25)	52.5 % (21)	0.033
Injury Severity Score (ISS) - mean (SD)	5.30 (8.46)	3.53 (9.62)	5.18 (10.65)	7.95 (6.68)	7.65 (9.16)	0.009
S-etanol in mmol/l, intoxicated pt - mean (SD)	44.82 (20.66)	34.34 (26.81)	53.73 (6.04)	51.67 (13.00)	23.83 (16.13)	0.081
Intoxication on admission % (*n*)	8.6 % (12)	4.8 % (10)	7.1 % (4)	16.2 % (6)	17.6 % (6)	0.031
Admission to intensive care unit (ICU) % (*n*)	36.4 % (56)	30.1 (69)	41.7 % (25)	42.5 % (17)	42.5 % (17)	0.23

**Table 4:** Patient characteristics at primary vs tertiary hospital. After subdivision into the studied groups

	Overall	Primary hospital	Tertiary hospital	*P*-value
	(*n* = 523)	(*n* = 250)	(*n* = 273)	
Age - mean, years (SD)	36.39 (20.74)	35.11 (20.58)	37.56 (20.86)	0.18
Male gender % (*n*):	65.0 % (340)	63.2 % (158)	66.7 % (182)	0.41
Examined with whole body computer tomography in trauma (WBCT-T) % (n)	55.4 % (290)	40.0 % (100)	69.6 % (190)	<0.001
Injury on WBCT-T % (*n*)	43.4 % (126)	45.0 % (45)	42.6 % (81)	0.7
Specific radiological exams % (*n*)	53.7 % (281)	60.0 % (150)	48.0 % (131)	0.006
Injury Severity Score - mean (SD)	4.89 (9.16)	4.79 (8.55)	4.99 (9.70)	0.8
S-etanol in mmol/l, intoxicated pt - mean (SD)	40.77 (21.52)	41.33 (26.83)	40.36 (17.37)	0.89
Intoxication on admission % (*n*)	8.0 % (38)	6.4 % (16)	9.8 % (22)	0.17
Highest level of care				<0.001
Admission to intensive care unit (ICU) % (*n*)	35.2 % (184)	58.0 % (145)	14.3 % (39)	
Admission to ward % (*n*)	19.9 % (104)	14.8 % (37)	24.6 % (67)	
Admission to clinical desicion unit (CDU) <24 h % (*n*)	10.7 % (56)	13.2 % (33)	8.5 % (23)	
Discharged from emergency department (E.D) % (*n*)	34.1 % (178)	14.0 % (35)	52.6 % (143)	

Of the total of 523 trauma alert patients, only 47 patients (9.0 %) did not go through any radiological exams. WBCT was performed in 290 patients (55.4 %), and showed traumatic findings in 125 patients (43.1 % of those examined with WBCT). Specific radiological imaging, alone or in addition to WBCT, were performed in 281 patients (53.7 %). Patients examined with WBCT in all risk groups, tabulated in Table 5, had no difference in age or sex, but there was a trend towards younger patients in the low risk group (*p* = 0.099).

**Table 5:** Patient characteristics in patients examined with whole body computer tomography in trauma (WBCT-T). After subdivision into the studied groups

	Low risk group	Intermediate risk group	High risk group	P-value
	(*n* = 45)	(*n* = 194)	(*n* = 51)	
Age - mean, years (SD)	32.73 (19.60)	39.42 (19.87)	36.09 (19.40)	0.1
Male gender % (*n*):	66.7 % (30)	70.6 % (137)	76.5 % (39)	0.56
Injury Severity Score (ISS)- mean (SD)	0.96 (1.02)	4.86 (7.17)	16.71 (17.72)	<0.001
Injury on WBCT-T % (*n*)	0 % (0)	44.8 % (87)	74.5 % (38)	<0.001

Overall, mean ISS was higher in patients examined with WBCT 6.33 (10.66 SD) compared to patients not examined with WBCT 3.12 (6.44 SD). In the low risk group there was no difference in ISS between patients examined with WBCT 1.15 (1.66 SD) and those who were not 0.81 (1.79 SD). At follow-up of 36 months or more, there was no finding of missed injuries. No injuries were found on any of the WBCTs performed in the low risk group, Table 5.

## Discussion

The use of WBCT is important in detecting injuries and evaluation of injury severity, which is of importance for planning treatment. It is also important in ruling out serious injuries. WBCT has changed the way trauma patients receive care. The benefit of WBCT is based on the fact that up to 22 % of patients may have missed injuries [11]. However, these findings are often based on studies performed in major trauma units in the United States with a mix of penetrating and blunt trauma, and with a relatively extensive burden of severe trauma [12]. Additionally, WBCT has not been proven to decrease trauma mortality [13].

The trauma panorama varies between hospitals, as well as between countries and regions. The mode and severity of trauma injuries must be taken into account when planning trauma care delivery. The two centers showed similarities regarding: routines for trauma care, trauma cohorts, type of injuries and frequency of traumatic findings from imaging. Hence patients presenting to the two trauma units included in the current study may be representative of the average trauma cohort in Sweden. With a mean ISS of 4.9 (9.17 SD), the benefit of WBCT may be less prominent than in patients with more severe injuries. Judicious interpretation of data is encouraged as data comes from the Scandinavian setting with modern cars, extensive safety equipment and few motorcyclists. Estimated road traffic death rate (per 100 000 population) is very low in Sweden, estimated to 3.0 in 2012 [14]. The World health organisation (WHO) Global status report on road safety 2013 showed estimated road traffic death rate to be 5 for China, 11 for USA, 19 for Russia, 32 for South Africa, 34 for Iran, 34 for Nigeria, 37 for Venezuela and 38 for Thailand. WHO noted that approximately 1.24 million deaths occurred on the world’s roads in 2010 [15].

WBCT was readily available and widely used. Use of WBCT in severely injured patients is important for planning of treatment while it can be questioned in the seemingly not injured where it is used mainly to permit early discharge from the ED. According to this finding, it could be safe to abstain from WBCT in high-energy trauma patients with no clinical signs of severe injury and no intoxication, especially considering that a majority of these patients are <33 years of age. They could be observed in the ED 2–4 h and re-examined before discharge without imaging.

Almost 1/3 of the patients in the low risk group were examined with WBCT and the ISS in those patients may have been artificially inflated because of clinically occult, but insignificant findings such as minor hematomas or swelling. The average ISS of 0.84 in the low-risk group suggests that artificial inflation of ISS has been limited.

The mechanisms of blunt trauma in this study is comparable to most of the western world but penetrating trauma is rare in this study and in the Scandinavian countries in general, even though it has shown a small incline in the last few years, especially in urban settings [16]. The mean age of this cohort was 36.4 years (20.7 SD). With a life expectancy of more than 82 years [17], radiation induced cancer is a risk that has to be taken into consideration when setting up algorithms for trauma care. This is especially true in the low risk group which was even younger (mean age of 32.8 years), and who benefited the least from WBCT.

The novelty of this study is in recognizing the absence of positive clinical findings in patients subject to high-energy trauma, indicating that these patients do not benefit from a WBCT, and this was valid both at a small trauma-unit, and a large trauma center.

The use of WBCT and 24/7 availability of skilled personnel for performing and interpreting WBCT is also associated with significant cost. The cost of WBCT is estimated at 600 Euro at the university hospital. However, this cost is negligible if the WBCT detects a significant injury, or if use of WBCT makes possible avoidance of intensive care unit admission for observation.

Motor vehicle crashes (MVC) constituted 44 % of all trauma in this cohort, and the majority of these patients were unharmed. Still, they were treated according to the trauma algorithm, and subject to WBCT in many cases. A revision of the trauma alarm criteria, which would enable downgrading of MVC without clinical sign of injury to a lower grade, could reduce unnecessary exposure to radiation. It has been reported that trauma alarm criteria can be safely limited with a reduction in overtriage from 51–29 % with only a slight increase in undertriage from 1–3 % [18]. Fall >3 m in height as mechanisms of injury was a risk factor for more severe injuries in this cohort, but patients in this group who met the “low risk” criteria still did not have any missed injuries.

The difference between the two hospitals in this study is mainly in admission rates. The smaller county hospital has a policy to observe trauma-patients in the ICU (58 %), whereas the university hospital has a more restrictive approach (14.3 %). No significant difference in the trauma patient population was seen between the hospitals.

### Limitations

Although the mechanisms of injury have been thoroughly evaluated at data collection, the retrospective nature of this study introduces a risk of bias. A prospective validation of the risk stratification criteria would increase the importance of our results. The material of 523 consecutive trauma patients is too small to compare mortality. No outcome follow-up such as the Glasgow outcome scale (GOS) was used.

## Conclusions

Risk stratification criteria can be used for deciding need of imaging in patients subject to high-energy trauma. WBCT does not affect patient care in high-energy trauma if the patient is mentally alert, not intoxicated nor shows signs of other than minor injuries when evaluated by a trauma-team. The risk of missing important traumatic findings in these patients is very low. Observation of the low-risk patient with re-examination instead of imaging may be considered in this group of often young patients where radiation exposure is an issue. After observation in the ED, most of these patients can be discharged without follow-up.

## References

[1] Bauer R, Steiner M, Kisser R, Macey SM, Thayer D (2014). Accidents and injuries in the EU. Results of the EuroSafe Reports. Bundesgesundheitsblatt Gesundheitsforschung Gesundheitsschutz.

[2] American College of Surgeons Comittee on Trauma. Advanced Trauma Life Support Student Course Manual. 2008.

[3] Baker SP ONB, Haddon W, Long WB (1974). The Injury severity score - a method for describing patients with multiple injuries and evaluating emergency care. J Trauma.

[4] Leidner B, Adiels M, Aspelin P, Gullstrand P, Wallen S (1998). Standardized CT examination of the multitraumatized patient. Eur Radiol.

[5] Leidner B, Adiels M, Aspelin P (1994). [Computer tomography. Life-threatening injuries of the skull, thorax, abdomen and pelvis are diagnosed in 15 minutes]. Lakartidningen.

[6] Hessmann MH, Hofmann A, Kreitner KF, Lott C, Rommens PM (2006). The benefit of multislice CT in the emergency room management of polytraumatized patients. Acta Chir Belg.

[7] Laack TA, Thompson KM, Kofler JM, Bellolio MF, Sawyer MD, Laack NN (2011). Comparison of trauma mortality and estimated cancer mortality from computed tomography during initial evaluation of intermediate-risk trauma patients. J Trauma.

[8] Corwin MT, Sheen L, Kuramoto A, Lamba R, Parthasarathy S, Holmes JF (2014). Utilization of a clinical prediction rule for abdominal-pelvic CT scans in patients with blunt abdominal trauma. Emerg Radiol.

[9] Kendall JL, Kestler AM, Whitaker KT, Adkisson MM, Haukoos JS (2011). Blunt abdominal trauma patients are at very low risk for intra-abdominal injury after emergency department observation. West J Emerg Med.

[10] Mahoney E, Agarwal S, Li B, Dechert T, Abbensetts J, Glantz A (2012). Evidence-based guidelines are equivalent to a liberal computed tomography scan protocol for initial patient evaluation but are associated with decreased computed tomography scan use, cost, and radiation exposure. J Trauma Acute Care Surg.

[11] Pfeifer R, Pape HC (2008). Missed injuries in trauma patients: A literature review. Patient Safety Surg.

[12] Fantus RJ, Nance ML (2015). NTDB data points: Annual Report 2014: How severe is it?. Bull Am Coll Surg.

[13] Ahmadinia K, Smucker JB, Nash CL, Vallier HA (2012). Radiation exposure has increased in trauma patients over time. J Trauma Acute Care Surg.

[14] OECD (2014). Road Safety Annual Report 2014.

[15] WHO. Global status report on road safety 2013: supporting a decade of action. Luxembourg 2013.

[16] SweTrau - Svenska traumaregistret 2014-12-02. Swedish trauma registry annual report (Årsrapport Swetrau 2014). Online at http://rcsyd.se/swetrau/wp-content/uploads/sites/10/2015/10/Årsrapport-SweTrau-2014_SKL.pdf. Accessed 02 Dec 2014.

[17] Anell A, Glenngard AH, Merkur S (2012). Sweden health system review. Health Syst Transit.

[18] Lehmann RK, Arthurs ZM, Cuadrado DG, Casey LE, Beekley AC, Martin MJ (2007). Trauma team activation: simplified criteria safely reduces overtriage. Am J Surg.

